# Rapid, contamination-less, and efficient environmental DNA filtration system^✰^

**DOI:** 10.1016/j.mex.2024.102621

**Published:** 2024-02-24

**Authors:** Takao Yoshida, Aya Yamazaki, Masaru Kawato, Yoshihiro Fujiwara

**Affiliations:** Marine Biodiversity and Environmental Assessment Research Center (BioEnv), Research Institute for Global Change (RIGC), Japan Agency for Marine-Earth Science and Technology (JAMSTEC), 2-15 Natsushima-cho, Yokosuka 237-0061, Japan

**Keywords:** eDNA, Deep-sea water, Biodiversity monitoring, Scarcely contaminated filtration, Large volume and multiple filtration system for environmental DNA

## Abstract

Due to the sporadic distribution and trace amount of environmental DNA (eDNA) in deep-sea water, in the context of biodiversity monitoring, large volumes of filtration and multiple filtration replicates are required for eDNA metabarcoding. To address issues tied to the use of multiple filtration devices and large filtration volumes (e.g., contamination, time consumption, etc.), we have developed two systems for simple, rapid, and contamination-less filtration simultaneously that allow for the processing of multiple sample replicates from large volumes of water. First, the water from a Niskin bottle was filtered directly using a solenoid pump. Second, the pumped deep-sea water, using the siphon effect, was directly filtered by a filtration device driven by water pressure. This system can process 24 replicates simultaneously without the need for expensive equipment and active driving force. Compared with conventional filtering methods, e.g., peristaltic pumps, the proposed systems reduce filtration time, minimizing contamination, and enabling the simultaneous acquisition of multiple replicates. Overall, the systems presented here provide an effective approach for eDNA metabarcoding analysis, particularly for the filtration of large volumes of water containing small amounts of eDNA, such as deep-sea water.

• The present systems reduce filtration time and contamination without water having to be transferred.
• Simultaneous multiple replicates improve the efficiency and reliability of biodiversity assessments.

## Graphical abstract

**Figure:**
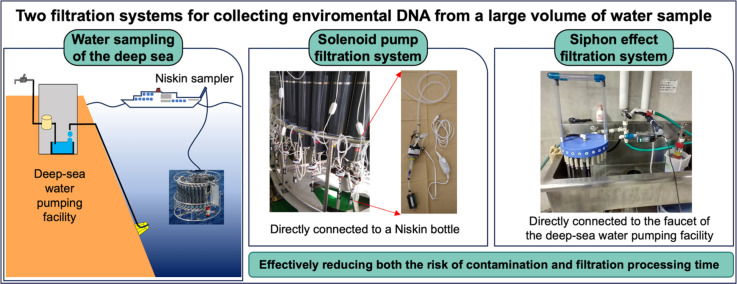

Specifications table

**Table:** 

Subject area:	Environmental Science
More specific subject area:	Environmental DNA
Name of your method:	Large volume and multiple filtration system for environmental DNA
Name and reference of original method:	T. Yoshida, M. Kawato, Y. Fujiwara, Y. Nagano, S. Tsuchida, A. Yabuki, Optimization of environmental DNA analysis using pumped deep-sea water for the monitoring of fish biodiversity, Front. Mar. Sci. 9 (2023), doi: 10.3389/fmars.2022.945758.
Resource availability:	Not applicable.

**Method details**

## Background

Environmental DNA (eDNA) metabarcoding is an effective method for detecting the presence of organisms and monitoring biodiversity in aquatic environments, including the deep sea. The filtration process used to collect eDNA from the water samples is essential for eDNA analysis. eDNA is sporadically distributed [1], [2], [3], and in deep-sea water, it is present in trace amounts owing to the lower organism density compared to shallow waters [4]. Consequently, effective biodiversity monitoring in deep-sea environments requires both a large volume of filtration (e.g., 10–20 L per filter) and multiple filtration replicates (e.g., up to 10) for an accurate assessment of biodiversity in surveyed habitats [5,6]. This highlights the significance of new filtration techniques.

Water sampling in the deep sea is generally conducted using multiple Niskin bottles (e.g., 12-L volume bottles) equipped with CTD devices. Recently, eDNA in pumped deep-sea water has been examined for the continuous monitoring of deep-sea fish communities [5]. However, analyzing eDNA from deep-sea water presents several challenges. Before filtration using conventional peristaltic pumps and aspirators [7], the collected water must be meticulously transferred to clean, eDNA-free containers like plastic bags to minimize contamination. Immediate filtration is imperative to prevent eDNA degradation [8,9], necessitating a clean working environment and adherence to sterile procedures to mitigate contamination risks [10]. Moreover, conventional peristaltic pumps and aspirators have limitations in filtration time for processing large water volumes [5] and in the number of samples that can be processed simultaneously, posing significant constraints. These pumps are costly, and even when using a manifold system with increased outlet filtration lines to accommodate multiple sample filtration, they may lack sufficient pressure for effective filtration. Conducting multiple filtration operations simultaneously demands a considerable number of pumps, leading to escalated costs. To address these challenges, our study introduces two low-cost filtration systems capable of simultaneously filtering multiple replicates, offering straightforward, rapid, and contamination-free processing. Consequently, these systems are suitable for efficiently processing large volumes of water. Moreover, their applicability extends beyond deep-sea water filtration to various types of facilities and containers.

### Solenoid pump filtration system

**Fig. 1:**
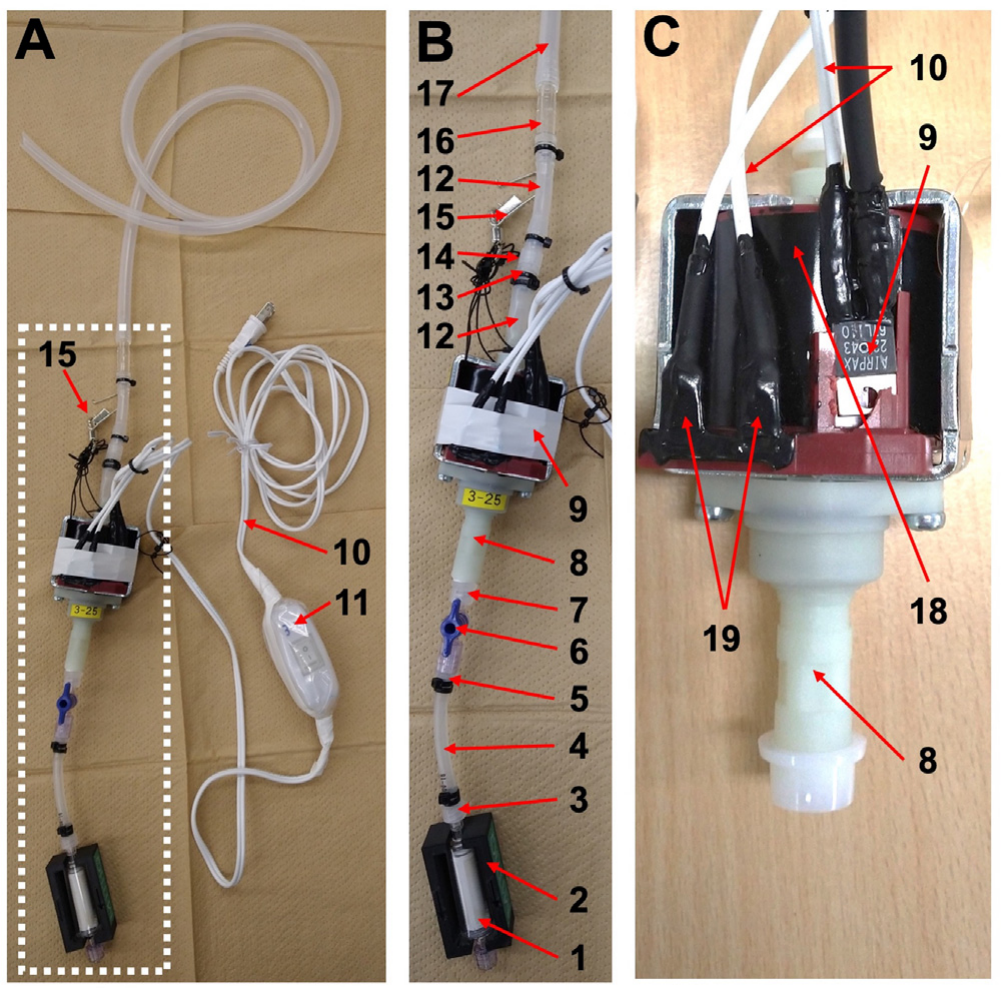
Assembled solenoid pump filtration system. Assembled solenoid pump filtration system (A). Magnified assembled solenoid pump (B) in the area within the dotted square in (A). Solenoid pump with thermostat (C). 1: Filter cartridge; 2: Filter cartridge cover; 3: Male Luer adaptor to hose barb adaptor (Outer diameter: 4 mm); 4: Silicon tube 1 (Inner diameter: 3.1 mm; outer diameter: 6.5 mm; maximum pressure: 270 kPa); 5: Female Luer adaptor to hose barb adaptor (Outer diameter: 4 mm); 6: Two way Luer stop cock; 7: Male Luer adaptor to 1/8–27NPT thread; 8: Solenoid pump; 9: Thermostat; 10: Power code with plug; 11: Plastic bag covered power switch; 12: Silicon tube 2 (Inner diameter: 6 mm; outer diameter: 8 mm); 13: Female Luer adaptor to hose barb adaptor (Outer diameter: 6.5 mm); 14: Male Luer adaptor to hose barb adaptor (Outer diameter: 6.5 mm); 15: Hook; 16: Hose barb joint; 17: Silicon Tube 3 (Inner diameter: 7 mm; outer diameter: 10 mm); 18: Heat-conductive adhesive sheet; 19: Plug of solenoid pump.

**Fig. 2:**
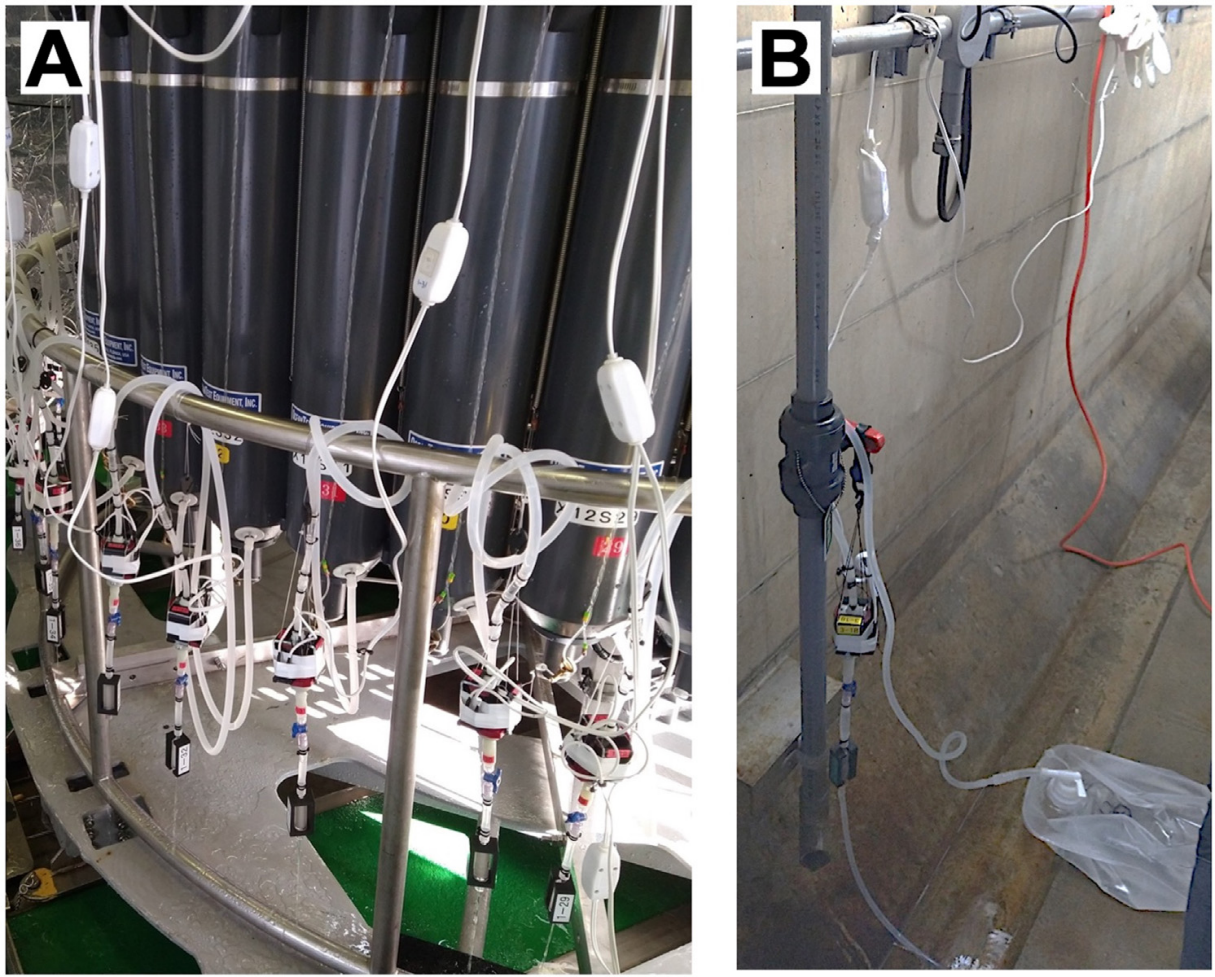
Filtering of water using solenoid pump filtration system. Filtration from Niskin bottles during the MR-23–03 research cruise (A) and plastic bags at the deep-sea water pumping facility in Yaizu (B).

This system employs a solenoid pump, commonly found in coffee espresso machines, for direct water filtration from Niskin bottles. It comprises a solenoid pump connected to a filter cartridge protected by a custom-made cover to prevent damage from pump pressure. This setup is linked to the Niskin bottle via tubing, a male Luer lock fitting, and a hose barb connector (as illustrated in Fig. 1, Fig. 2A), eliminating the need for water transfer to a separate container. Detailed components of the system are depicted in Fig. 1 and outlined in Table 1. With a total cost of approximately 54 USD per piece, excluding the filter cartridge, this system offers a more cost-effective and efficient alternative to conventional pumps like peristaltic pumps. Multiple solenoid pumps can be used simultaneously to facilitate the filtration of large water volumes.

**Table 1:** List of components in the solenoid pump system and the faucet filtration system.

System	Item No.	Component
The solenoid pump system		

	1	Filter cartridge (Sterivex filter cartridge, Merck Millipore, Darmstadt, Germany; pore size, 0.45 µm; maximum inlet pressure: 310 kPa)
	2	Filter cartridge cover (self-made)
	3	Male Luer adaptor to hose barb adaptor (Outer diameter: 4 mm)
	4	Silicon tube 1 (Inner diameter: 3.1 mm; outer diameter: 6.5 mm; maximum pressure: 270 kPa)
	5	Female Luer adaptor to hose barb adaptor (Outer diameter: 4 mm)
	6	Two-way Luer stop cock
	7	Male Luer adaptor to 1/8–27NPT thread
	8	Solenoide pump (KP1, Cnkalun, Dongguan Yanlun Electric Co.,Ltd, China; Power: AC110–120 V, 35 W; maximum pressure: 1.5 MPa; maximum flow rate: 750 ml/min)
	9	Thermostat (Opening temperature: 110 °C; closing temperature: 80 °C)
	10	Power code with plug
	11	Power switch
	12	Silicon tube 2 (Inner diameter: 6 mm; outer diameter: 8 mm)
	13	Female Luer adaptor to hose barb adaptor (Outer diameter: 6.5 mm)
	14	Male Luer adaptor to hose barb adaptor (Outer diameter: 6.5 mm)
	15	Hook
	16	Hose barb joint (Connection tube inner diameter: 5 to 9 mm)
	17	Silicon tube 3 (Inner diameter: 7 mm; outer diameter: 10 mm)
	18	Heat-conductive adhesive sheet (HF-C1225, Sunhayato corp., Tokyo, Japan)

The faucet filtration system		

	1	Filtering device (TK-MC-300–40–24–1/8PT, Takizawa factory, Yokosuka, Japan; Diameter: 30 cm; thickness: 4 cm, 24 filtration channels)
	2	Detachable flexible nylon tube (Inner diameter: 11 mm; outer diameter: 16 mm)
	3	One-touch fitting connection (R1/2 thread)
	4	Connector (Rp1/2 thread)
	5	Polyvinyl chloride pipe (Inner diameter: 16 mm; outer diameter: 22 mm)
	6	Flow meter (FD-H20, KEYENCE, Osaka, Japan)
	7	Male Luer adaptor to 1/8–27NPT thread
	8	Two-way Luer stop cock
	9	Pre-filter (VYS4UUENJ015, ASAHI AV, Tokyo, Japan)
	10	Filter cartridge (Sterivex filter cartridge, Merck Millipore, Darmstadt, Germany; pore size, 0.45 µm; maximum inlet pressure: 310 kPa)
	11	Stopped screws (RC1/4–19)
	12	Stopped screw (RC1/2–14)

The structure of the solenoid pump filtration system is as follows:

• The solenoid pump was covered with a heat-conductive adhesive sheet (HF-C1225, Sunhayato corp., Tokyo, Japan) and equipped with a thermostat to prevent overheating (Fig. 1C).
• The power switch, thermostat, and solenoid pump were soldered together, and the connections were insulated using heat-shrink tubing and rubber coating (Fig. 1A, B and C).
• The power switch was covered with a plastic bag for waterproofing (Fig. 1A).
• The male thread (1/8–27NPT) of the male Luer adaptor (Component No.7 in Table 1) was hand-cut to fit an R1/8 screw, as the outlet of the solenoid pump had a GAS 1/8 thread and then connected to the solenoid pump (Fig. 1B).
• The hook was attached to the solenoid pump using a beading thread (Fig. 1A and B).
• Luer adaptors or hose barb joints were connected to the tubes, and the connections between joints and tubes were tightly secured using an insulation lock (Fig. 1B). These components were assembled around a solenoid pump coupled with a two-way Luer stopcock (Fig. 1B).
• A self-made cover was utilized to safeguard the filter cartridge against the potential disassembly of the filter cartridge under pressure (Fig. 1B).

Filtration using this system was proceeded as follows (as shown in Fig. 4):

• Disposable laboratory rubber gloves should always be worn during subsequent procedures to prevent contamination. If gloves were contaminated during work, they were immediately replaced.
• The system was sterilized by filling and continuously flowing the lines with 2 L of 2% chlorine-based commercial bleach (Kao Co., Tokyo, Japan) through the lines using a solenoid pump for at least 5 min. After sterilization, the lines were thoroughly rinsed with 2 L of pure distilled water (Milli-Q water, Millipore). The sterilized system was stored in a clean plastic bag until use.
• A solenoid pump is attached to the CTD frame using a hook, as shown in Fig. 2A, and the plug is connected to a power source.
• Before filtration, the outlet of the Niskin bottle was wiped with clean paper to prevent contamination of the working area. The outlet of the Niskin bottle was connected to the inlet of the sterilized tube.
• An assembled filter cartridge with a cover was connected to the outlet of the solenoid pump line, and filtration was initiated.
• If the buffer tube (as shown in Fig. 1A, No.4) upstream of the filter cartridge had expanded during filtration, a two-way Luer stopcock was employed (Fig. 1B, No.6) to control the flow rate. This step was necessary as the maximum pressure resistance of the tube (270 kPa) is lower than that of the filter cartridge (310 kPa). Therefore, the buffer tube method was used to regulate the filtration pressure.
• After filtration, the lines were thoroughly rinsed with pure distilled water or fresh water using a pump to remove seawater, and the system was stored in a clean plastic bag.
• The solenoid pump should be handled with caution due to its extremely high temperature during filtration.

This system was directly connected to the outlet of a container including a Niskin bottle, eliminating the need to transfer water from the collection bottle (e.g., Niskin bottle) to separate containers, a process prone to contamination. This direct connection facilitates swift and uncomplicated filtration, effectively reducing the likelihood of contamination.

### Faucet filtration system

The deep-sea water pumping facility comprises a deep-sea water intake, pipeline, and pipeline outlet situated approximately 10 m below the sea surface. In this setting, water naturally flows out of the pipeline outlet due to pressure created by the height difference, utilizing the siphoning effect without requiring additional energy. A faucet was installed at the outlet of a pipeline in a deep-sea water pumping facility. This system was directly connected to the faucet at the outlet of the pipeline for filtration purposes. Consequently, the pressure of the deep-sea water from the faucet measured approximately 100 kPa, which is lower than the maximum inlet pressure resistance of the filter cartridge. Previously, we developed a faucet filtration system involving the attachment of 18 filter cartridges and the measurement of filtration volume at the outlet of each filter cartridge [5]. In this study, we developed a new system in which the filtration volume is measured upstream of the filtering device.

**Fig. 3:**
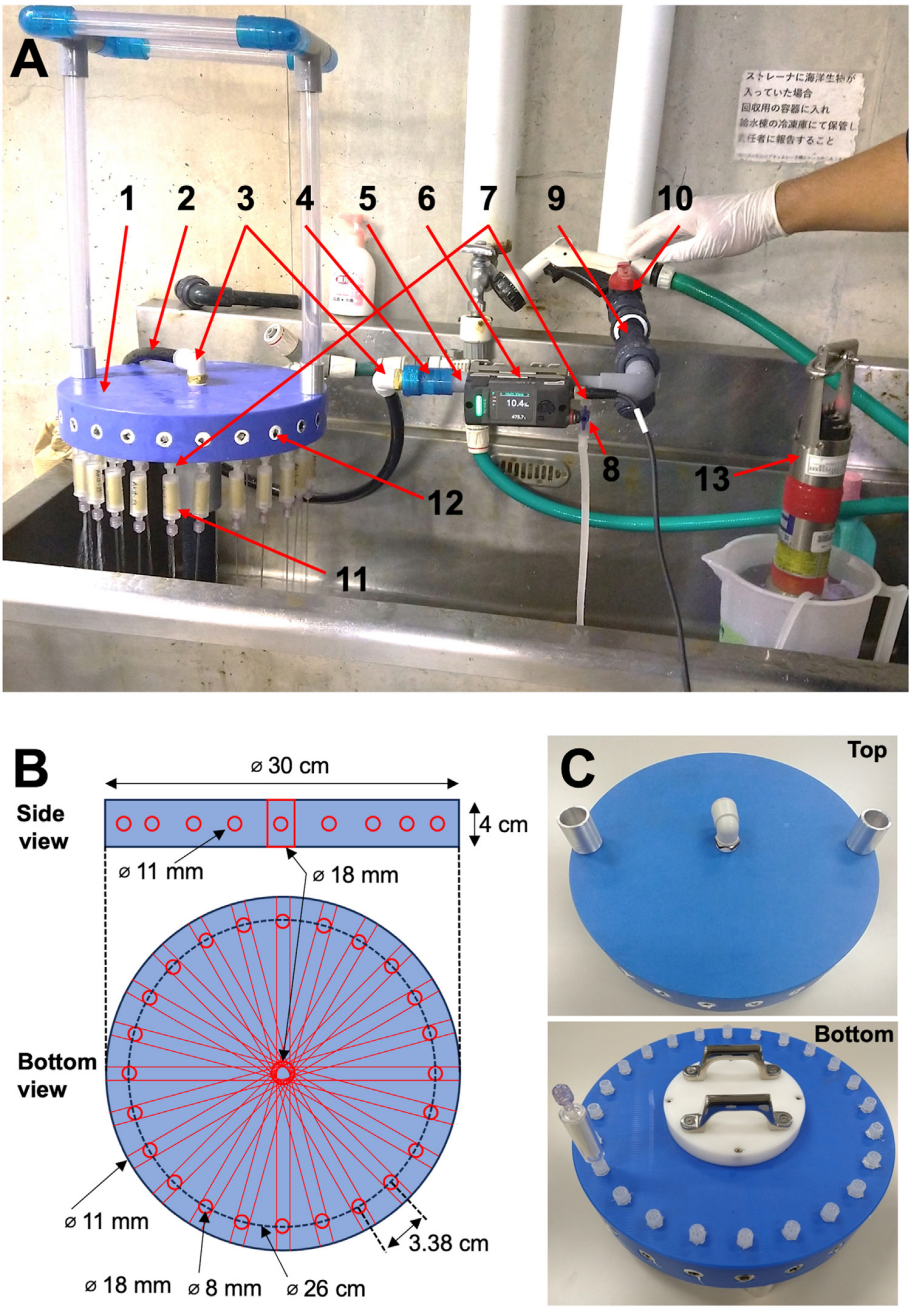
Assembled faucet filtration system. Assembled faucet filtration system in Akazawa (A). 1: Filtering device; 2: Nylon tube; 3: One-touch fitting connection; 4: Connector (Rp1/2 thread); 5: Polyvinyl chloride pipe; 6: Flowmeter; 7: Male Luer adaptor to 1/8–27NPT thread; 8: Two-way stop cock; 9: Pre-filter; 10: Faucet in deep-sea water pumping facility; 11: Filter cartridge; 12: stopped screw; 13: Rinko profiler sensor. Schematic of the filtering device (B). The flow lines in the filtering device created by drilling the holes are shown as red lines and circles. Filtration device (C). The upper and lower panels show the top and bottom parts of the filtering device, respectively.

The system consisted of a filtering device with 24 channels for filter cartridges, pipes, a pre-filter, and a flow meter (FD-H20, KEYENCE, Osaka, Japan) (Fig. 3). The lengths of the flow lines from the inlet to the outlet in the filtering device were kept identical, ensuring an equal water flow rate through each filter cartridge. Consequently, the water volume filtered through each cartridge remained consistent. A flow meter was easily attached by clamping it to the outside of the pipe at the upstream end of the filtering device, allowing for the measurement of the total flow volume. Thus, the filtration volume for each filter cartridge was determined by calculating the total flow volume and dividing it by the total number of filters. To monitor the physico-chemical parameters of the water (such as pH, temperature, conductivity, salinity, turbidity, and dissolved oxygen concentration) using a measuring device (Rinko Profiler Sensor, JFE Advantech Co., Ltd., Nishinomiya, Japan), a two-way stopcock was installed upstream of the flow sensor without interfering with its operation. The details of the components used in this system are presented in Fig. 3 and Table 1.

The structure of the faucet filtration system is as follows:

• The filtering device was made of monomer-cast nylon and formed a circular disk (diameter: 30 cm; thickness: 4 cm).
• The flow lines of the filtering device were created by drilling holes 11 mm in diameter from the side of the device (Fig. 3B). Stopped screws (RC1/4–19) (Component No.11 in Table 1) were then connected to the exteriors of these holes, as shown in Fig. 3A and B.
• The flow line outlets of the filtering device were created by drilling holes with diameters of 8 mm, as shown in Fig. 3B. The male thread (1/8–27NPT) of the male Luer adaptor was hand-cut to fit an R1/8 screw, and then the male Luer adaptors were connected to the flow line outlets of the filtering device, as shown in the lower panels of Fig. 3B and C.
• A flow line inlet for the filtering device was created by drilling a hole with a diameter of 18 mm (Fig. 3B). A one-touch fitting connection was attached to the flow line inlet at the top of the filtering devices, as shown in the upper panels of Fig. 3A and C. A stopped screw (RC1/2-14) (component 12 in Table 1) was connected to the bottom of the filtering device. The handles were attached to the upper part of the stopped screw at the bottom of the filtering device, as shown in Fig. 3C lower panel.
• A polyvinyl chloride pipe, pre-filter, and another one-touch fitting connection were assembled as shown in Fig. 3A.
• The flowmeter was fixed by clamping it outside the pipe, as shown in Fig. 3A.
• To monitor water conditions such as pH, temperature, and dissolved oxygen concentration using the Rinko Profiler, a dedicated flow line was established. This involved drilling an 8 mm diameter hole in the pipe upstream of the flow meter. Subsequently, a male Luer adaptor, hand-cut to fit an R1/8 screw, was connected to the drilled hole. A two-way Luer stopcock was then connected using a male Luer adaptor, as shown in Fig. 3A.
• A detachable flexible nylon tube was attached using one-touch fitting connections between the filtering device and pipeline, as shown in Fig. 3A.
• The total length of the flow line from the inlet to the outlet was approximately 185 cm.

**Fig. 4:**
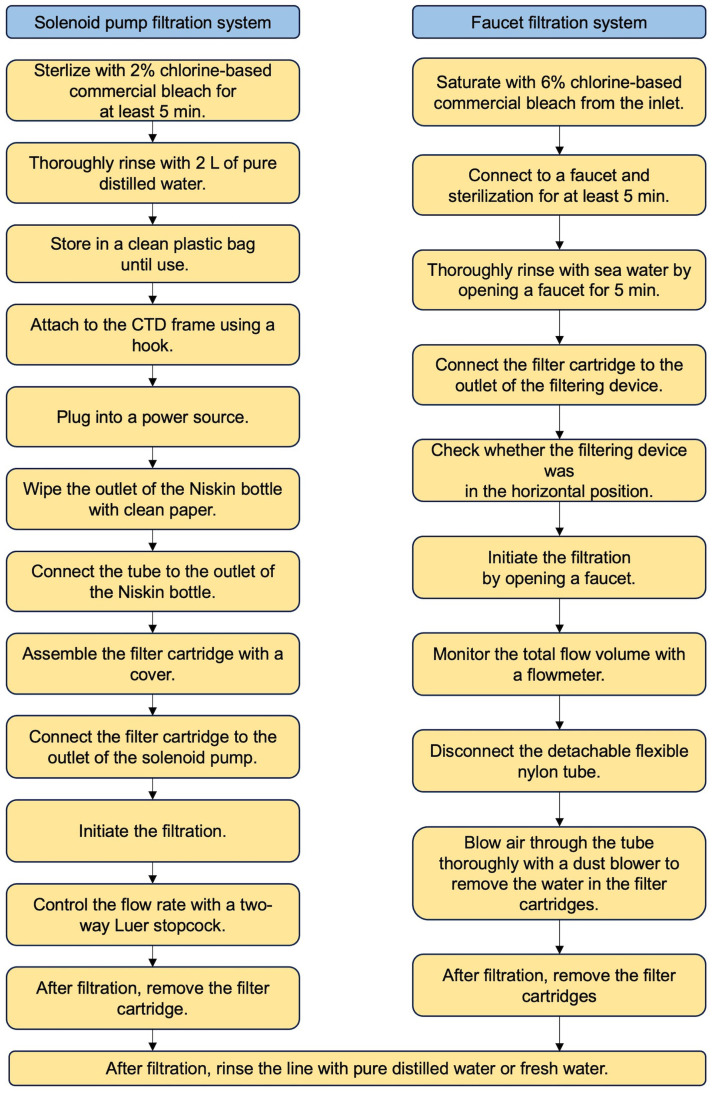
Filtration protocols of the solenoid pump system and the faucet filtration system.

Filtration using this system was performed as follows (as shown in Fig. 4):

• Disposable laboratory rubber gloves should always be worn during subsequent procedures to prevent contamination. If gloves were contaminated during work, they were immediately replaced.
• Sterilization of the system was conducted at the pumping facility just before filtration. This process involved saturating the lines with a 6% chlorine-based commercial bleach from the inlet and promptly connecting them to the faucet in the deep-sea water pumping facility. Subsequently, the system underwent thorough sterilization for at least 5 min. Deep-sea water was then flushed through the lines by opening the faucet. The faucet was closed after washing the lines for approximately 5 min.
• The filter cartridges were connected to the outlet of the filtering device.
• After checking whether the filtering device was in the horizontal position, filtration was initiated by opening the faucet.
• The total amount of water passing through the piping was monitored using a flowmeter.
• After filtration, the detachable flexible nylon tube was disconnected from the line below the flow meter, and air was blown through the tube using a dust blower (GSA01Z, Makita Corp., Aichi, Japan) to remove water from the filter cartridge. The filter cartridges were removed from the filtering device.
• The lines were thoroughly rinsed with pure distilled water or fresh water to remove seawater, and the system was stored in a clean plastic bag.

Similar to the solenoid pump filtration system, this system is directly connected to faucets, whether on ships, land, or the pumped deep-sea water facility. This direct connection eliminates the necessity of water transfer, which is linked with contamination. Consequently, it facilitates swift and straightforward filtration, significantly reducing the risk of contamination.

## Method validation

### Filtration using the solenoid pump filtration system

We conducted a filtration test of deep-sea water using a solenoid pump filtration system at a deep-sea water pumping facility in Yaizu, Shizuoka Prefecture, on the Pacific coast of central Japan facing Suruga Bay, from March 7 to 8, 2023, utilizing a solenoid pump filtration system. The intake depth for pumping deep-sea water was 400 m. During the test, we compared the filtration efficiency of the solenoid pump with that of a peristaltic pump. Each condition involved filtering at least three replicates of 10 liters of deep-sea water (as shown in Fig. 2B). The mean of filtration time using the solenoid pump was approximately 35 min, while with the peristaltic pump, it averaged around 40 min, consistent with a previous report [5]. To assess the efficiency of eDNA filtration by the solenoid pump, we conducted eDNA analysis of fish species using the “MiFish” fish universal primers [11]. To compare the fish species detected using both the solenoid pump and peristaltic pump filtration methods, we examined a total of four filters under the same conditions over two days (March 7 and 8, 2023). Two filters were processed using solenoid pumps, while the remaining two underwent filtration with a peristaltic pump. Each day, one filtration replicate was conducted using each pump. Subsequently, eDNA was extracted from the filter cartridges, and library construction and sequencing were performed using an updated method [12] based on a previous study [11].This method involves several key steps. Initially, the filter cartridge is opened, and the filter is shredded, followed by its incubation in a microtube. This process enhances the efficient lysis of the environmental DNA source [12]. Library preparation including two-step PCR for metabarcoding of fish eDNA was performed [12]. In the first PCR, the Platinum SuperFi II DNA Polymerase demonstrates efficacy in amplifying the fish target region of the mitochondrial 12S rRNA gene sequence, utilizing two universal primer sets: MiFish-U-Forward and Reverse, and MiFish-E-Forward and Reverse [11] from trace amounts of extracted eDNA [12]. Following the initial PCR, the subsequent steps involve a second round of PCR and next-generation sequencing, carried out by Bioengineering Lab. Co., Ltd (Sagamihara, Japan). The resulting sequences were then assembled and subjected to taxonomic assignment as described previously [5]. On March 7, 41 fish species were detected in the filters using the solenoid pump, and 37 were detected in the peristaltic pump. On March 8, 41 fish species were detected in the filters using the solenoid pump, and 44 in those using the peristaltic pump. Moreover, there was no indication of human-derived contamination in any of the filters examined. Given the sporadic distribution of eDNA sources in seawater [1], [2], [3], the detection of species using eDNA is significantly influenced by this sporadicity [5]. Consistent with previous findings showing variability in the number of fish species detected from filters collected under identical water conditions in Yaizu [5], this study is expected to reveal similar variability. These differences in species detection are likely attributable to these inherent challenges. However, PERMANOVA revealed no significant differences in fish community compositions between the solenoid and peristaltic pump methods (PERMANOVA, R^2^ = 0.113, *F* = 0.734, *p* = 0.606). This finding suggests that, despite variations in the number of detected species, the overall performance of the solenoid pump filtration system is comparable to that of the peristaltic pump. Consequently, the solenoid pump filtration system emerges as an efficient and rapid alternative for eDNA collection, delivering a performance similar to that of the conventional peristaltic pump method.

During the MR-23–03 research cruise in May 2023, we collected deep-sea water from 36 Niskin bottles installed in a CTD device, and filtration was executed by attaching one solenoid pump to each Niskin bottle as part of the field validation (Fig. 2A). A total of nine CTD casts, resulting in nine filtration rounds, were carried out in Suruga Bay, with 36 solenoid pumps utilized in a single CTD cast. Deep-sea water was collected at various depths (100, 400, 500, 100, 1500, and 2000 m) using 36 Niskin bottles (12 L per bottle) per one CTD cast. After retrieving the CTD onboard, during the fastest filtration process, the total filtration time, including attachment of the solenoid pump to the 36 Niskin bottles, was approximately 85 min. The previous method used three peristaltic pumps, each performing filtration through four filters simultaneously, which required approximately 180 min across three filtration rounds, excluding water transfer from the Niskin bottles to separate containers. Remarkably, this new process is approximately twice as fast as the previous conventional method. These results demonstrate the efficiency and effectiveness of the solenoid pump filtration system for eDNA analysis, particularly during research cruises and field research with an electric power supply. Overall, the proposed system exhibits great operational efficiency and minimizes contamination without requiring the transfer of water samples into separate containers.

### Filtration using the faucet filtration system

Filtration using the faucet filtration system was performed at a deep-sea water pumping facility in Akazawa, Shizuoka Prefecture, on the Pacific coast of central Japan facing Sagami Bay (Fig. 3A). At this facility, deep-sea water was pumped from a depth of 800 m, with the faucet positioned roughly 10 m below the sea surface. With the newly developed system, filtering 20 L of deep-sea water took approximately 35 min. The entire filtration process, which included system setup and two filtration rounds totaling 48 filter replicates, took approximately 120 min. This process was twice as fast as the previous system, which required three filtering cycles using 18 filter cartridges, consuming approximately four hours to process 48 filter replicates. The enhanced filtration method outlined here has the potential to significantly impact eDNA filtration efforts.

## Conclusion

In conventional filtration using a peristaltic pump, the operation involves rollers compressing a tube to drive fluid through it, necessitating frequent tube replacements. However, our newly developed filtration system eliminates the need for tube replacements, thereby markedly reducing operational costs and aligning with Sustainable Development Goals. Furthermore, it streamlines eDNA workflow by eliminating the need to transfer water samples to separate containers. This not only reduces contamination risks but also streamlines processing time and effort during filtration compared to conventional filtration methods. Our newly developed filtration systems are designed to seamlessly integrate with Niskin bottles, and faucets at a deep-sea water pumping facilities, offering versatility across various facilities and containers. This advancement significantly improves the efficiency of both multiple filtration replicates and the filtration of large volumes of water for eDNA analysis.

## CRediT authorship contribution statement

**Takao Yoshida:** Conceptualization, Methodology, Validation, Investigation, Writing – original draft, Writing – review & editing. **Aya Yamazaki:** Validation, Investigation, Writing – review & editing. **Masaru Kawato:** Investigation, Writing – review & editing. **Yoshihiro Fujiwara:** Conceptualization, Writing – review & editing.

## Declaration of competing interests

The authors declare that they have no competing financial interests or personal relationships that may have influenced the work reported in this study.

## Data Availability

Data will be made available on request.

## References

[1] Ahn H., Asano Y., Suzuki S., Ooki A. (2022). Positive effect of filtration additives for increasing environmental DNA detections in summer and winter oceanic waters. Environ. DNA.

[2] Bessey C., Jarman S.N., Berry O., Olsen Y.S., Bunce M., Simpson T., Power M., McLaughlin J., Edgar G.J., Keesing J. (2020). Maximizing fish detection with eDNA metabarcoding. Environ. DNA.

[3] Stauffer S., Jucker M., Keggin T., Marques V., Andrello M., Bessudo S., Cheutin M.C., Borrero-Pérez G.H., Richards E., Dejean T., Hocdé R., Juhel J.B., Ladino F., Letessier T.B., Loiseau N., Maire E., Mouillot D., Mutis Martinezguerra M., Manel S., Polanco Fernández A., Valentini A., Velez L., Albouy C., Pellissier L., Waldock C. (2021). How many replicates to accurately estimate fish biodiversity using environmental DNA on coral reefs?. Eco. Evol..

[4] McClenaghan B., Fahner N., Cote D., Chawarski J., McCarthy A., Rajabi H., Singer G., Hajibabaei M. (2020). Harnessing the power of eDNA metabarcoding for the detection of deep-sea fishes. PLoS ONE.

[5] Yoshida T., Kawato M., Fujiwara Y., Nagano Y., Tsuchida S., Yabuki A. (2023). Optimization of environmental DNA analysis using pumped deep-sea water for the monitoring of fish biodiversity. Front. Mar. Sci..

[6] Fujiwara Y., Tsuchida S., Kawato M., Masuda K., Sakaguchi S.O., Sado T., Miya M., Yoshida T. (2022). Detection of the largest deep-sea-endemic teleost fish at depths of over 2,000m through a combination of eDNA metabarcoding and baited camera observations. Front. Mar. Sci..

[7] Miya M., Minamoto T., Yamanaka H., Oka S.I., Sato K., Yamamoto S., Sado T., Doi H. (2016). Use of a filter cartridge for filtration of water samples and extraction of environmental DNA. J. Vis. Exp..

[8] Goldberg C.S., Turner C.R., Deiner K., Klymus K.E., Thomsen P.F., Murphy M.A., Spear S.F., McKee A., Oyler-McCance S.J., Cornman R.S., Laramie M.B., Mahon A.R., Lance R.F., Pilliod D.S., Strickler K.M., Waits L.P., Fremier A.K., Takahara T., Herder J.E., Taberlet P. (2016). Critical considerations for the application of environmental DNA methods to detect aquatic species. Methods Ecol. Evol..

[9] Holman L.E., Chng Y., Rius M. (2022). How does eDNA decay affect metabarcoding experiments?. Environ. DNA.

[10] Ruppert K.M., Kline R.J., Rahman M.S. (2019). Past, present, and future perspectives of environmental DNA (eDNA) metabarcoding: a systematic review in methods, monitoring, and applications of global eDNA. Global Ecol. Conser..

[11] Miya M., Sato Y., Fukunaga T., Sado T., Poulsen J.Y., Sato K., Minamoto T., Yamamoto S., Yamanaka H., Araki H., Kondoh M., Iwasaki W. (2015). MiFish, a set of universal PCR primers for metabarcoding environmental DNA from fishes: detection of more than 230 subtropical marine species. R. Soc. Open Sci..

[12] Kawato M., Yoshida T., Miya M., Tsuchida S., Nagano Y., Nomura M., Yabuki A., Fujiwara Y., Fujikura K. (2021). Optimization of environmental DNA extraction and amplification methods for metabarcoding of deep-sea fish. MethodsX.

